# Synergistic efficacy of inhibiting MYCN and mTOR signaling against neuroblastoma

**DOI:** 10.1186/s12885-021-08782-9

**Published:** 2021-09-26

**Authors:** Matthew J. Kling, Connor N. Griggs, Erin M. McIntyre, Gracey Alexander, Sutapa Ray, Kishore B. Challagundla, Shantaram S. Joshi, Don W. Coulter, Nagendra K. Chaturvedi

**Affiliations:** 1 Department of Pediatrics, Hematology/Oncology Division, University of Nebraska Medical Center, 986395, Nebraska Medical Center, Omaha, NE USA; 2 Department of Biochemistry and Molecular Biology, University of Nebraska Medical Center, Omaha, NE USA; 3 Department of Genetics, Cell Biology and Anatomy, University of Nebraska Medical Center, Omaha, NE USA

**Keywords:** Neuroblastoma, MYCN protein, mTOR signaling, Small molecule inhibitors

## Abstract

**Background:**

Neuroblastoma (NB) patients with MYCN amplification or overexpression respond poorly to current therapies and exhibit extremely poor clinical outcomes. PI3K-mTOR signaling-driven deregulation of protein synthesis is very common in NB and various other cancers that promote MYCN stabilization. In addition, both the MYCN and mTOR signaling axes can directly regulate a common translation pathway that leads to increased protein synthesis and cell proliferation. However, a strategy of concurrently targeting MYCN and mTOR signaling in NB remains unexplored. This study aimed to investigate the therapeutic potential of targeting dysregulated protein synthesis pathways by inhibiting the MYCN and mTOR pathways together in NB.

**Methods:**

Using small molecule/pharmacologic approaches, we evaluated the effects of combined inhibition of MYCN transcription and mTOR signaling on NB cell growth/survival and associated molecular mechanism(s) in NB cell lines. We used two well-established BET (bromodomain extra-terminal) protein inhibitors (JQ1, OTX-015), and a clinically relevant mTOR inhibitor, temsirolimus, to target MYCN transcription and mTOR signaling, respectively. The single agent and combined efficacies of these inhibitors on NB cell growth, apoptosis, cell cycle and neurospheres were assessed using MTT, Annexin-V, propidium-iodide staining and sphere assays, respectively. Effects of inhibitors on global protein synthesis were quantified using a fluorescence-based (FamAzide)-based protein synthesis assay. Further, we investigated the specificities of these inhibitors in targeting the associated pathways/molecules using western blot analyses.

**Results:**

Co-treatment of JQ1 or OTX-015 with temsirolimus synergistically suppressed NB cell growth/survival by inducing G1 cell cycle arrest and apoptosis with greatest efficacy in MYCN-amplified NB cells. Mechanistically, the co-treatment of JQ1 or OTX-015 with temsirolimus significantly downregulated the expression levels of phosphorylated 4EBP1/p70-S6K/eIF4E (mTOR components) and BRD4 (BET protein)/MYCN proteins. Further, this combination significantly inhibited global protein synthesis, compared to single agents. Our findings also demonstrated that both JQ1 and temsirolimus chemosensitized NB cells when tested in combination with cisplatin chemotherapy.

**Conclusions:**

Together, our findings demonstrate synergistic efficacy of JQ1 or OTX-015 and temsirolimus against MYCN-driven NB, by dual-inhibition of MYCN (targeting transcription) and mTOR (targeting translation). Additional preclinical evaluation is warranted to determine the clinical utility of targeted therapy for high-risk NB patients.

**Supplementary Information:**

The online version contains supplementary material available at 10.1186/s12885-021-08782-9.

## Background

Neuroblastoma (NB) is the most common extracranial pediatric solid tumor of neural crest origin and accounts for approximately 10% of childhood cancers and 15% of cancer-related deaths in children. Approximately 50% of NB patients are diagnosed with high-risk disease, and despite intensive multimodal therapy options (including radiation, surgery, and chemotherapy), effective treatment for these patients remains elusive [1, 2]. Particularly, amplification of the neural *MYC* (*MYCN)* oncogene, which occurs in 20–30% of all NB tumors and nearly 50% of the high- risk cases, remains a key predictor of poor outcomes. MYCN-amplified NB tumors typically exhibit high malignancy, metastatic properties, and treatment resistance [3, 4]. Therefore, upstream and downstream regulatory components of the MYCN-driven tumorigenic programs contain promising targets for the identification of novel therapeutics for these high-risk patients.

One of the most frequently deregulated oncogenic pathways in cancers, is the protein synthesis (translation) pathway that drives increased cell proliferation and cancer progression/resistance [5, 6]. Similar to MYC protein, MYCN plays an important role in protein synthesis by controlling the transcription of several components of protein synthesis machinery including components involved in mRNA translation and ribosome biogenesis [7–10]. Similar to MYC protein, MYCN itself is considered to be an undruggable target because of its short half-life and complex protein structure; however, targeting epigenetic regulators of MYCN provides a promising alternative strategy [11, 12]. Bromodomain and extra-terminal (BET) family proteins have been shown to promote MYCN transcription. In preclinical studies, inhibiting BET protein function has shown promise as a therapeutic strategy to target MYCN in NB and other cancers [13–17].

mTOR signaling is another key regulator of protein synthesis, which is frequently deregulated in cancers including NB [18–20]. MTOR kinase regulates protein synthesis by phosphorylating key translation factors (4EBP1/eIF4E) upstream of the translation initiation complex [18]. Notably, it has been shown that mTOR signaling can stabilize MYCN protein levels by inducing MYCN translation [21]. Together, these observations suggest the potential to block deregulated MYCN-driven proliferation by co-delivering drugs that target global transcription and translation.

We hypothesize that combined inhibition of transcription (by BET-protein inhibition) and translation (by mTOR inhibition) will synergistically blockade global protein synthesis and proliferation in MYC-driven NB tumor cells. Using small molecule/pharmacologic approaches, we tested this hypothesis by targeting BET with JQ1 or OTX-015 and mTOR with temsirolimus, in NB cell lines.

## Methods

### Cell lines and inhibitors

Non-MYCN-amplified NB cell lines (SK-K-AS, SK-N-SH) and MYCN-amplified NB cell lines (SK-N-BE2, IMR-32, and SK-N-DZ) were purchased from American Type Culture Collection (USA). Non-MYCN-amplified NB cell line CHLA-255 was provided by Dr. Kishore Challagundla (UNMC). The identity of cell lines was confirmed by their respective cell bank using STR analyses. Cell lines were also verified for mycoplasma-free condition using the MycoSensor-PCR assay kit (Agilent-Technologies, USA). Cell lines were cultured in Eagle’s Minimal Essential Medium (EMEM) or Roswell Park Memorial Institute (RPMI)-1640 media containing 10% fetal bovine serum and 1% penicillin-streptomycin (Invitrogen Life Technologies, USA). Experiments were performed under 8–10 passages for each cell line. Small molecule inhibitors (JQ1, OTX-015 and temsirolimus) and cisplatin (a chemotherapeutic drug) were purchased from Sellekchem LLC (USA).

### Cell viability assay

Effects of inhibitors on NB Cell growth/viability were assessed using the MTT assay as previously described [22, 23].

### Neurosphere assay

Effects of inhibitors on NB spheres were performed using the neurosphere/sphere assay as previously described [22].

### Cell cycle distribution and apoptosis analyses

Analysis of cell cycle distribution in NB cells was performed using a propidium iodide (PI staining) flow cytometry kit (Abcam, UK) according to manufacturer’s instructions. Apoptosis in NB cells was assessed using an Annexin-V flow-cytometry assay kit (BD-Biosciences, USA) following the manufacturer’s instructions. The flow cytometry analysis of Annexin-V/PI stained cells was determined using the FACSCalibur cell sorter system (BD Biosciences, USA).

### Global protein synthesis assay

NB cells were treated with inhibitors alone or in combination in 96-well plates (2 × 10^4^ cells/well) for 24 h. After treatment, culture media was replaced with fresh media containing O-propargyl-puromycin (OPP) and incubated for 30 min at 37 °C to be incorporated in translating polypeptide chains. Cells were then fluorescently stained with 5-FAM-Azide. The detection of fluorescent-labelled OPP was performed using the Protein Synthesis Assay Kit (#601100, Cayman Chemical, USA), according to the manufacturer’s instructions.

### Immunoblotting

Western blot analysis of the inhibitor-treated NB cells was performed as described previously [23]. Primary and secondary antibodies used in this analysis included MYCN (Cell Signaling Technology #9405), BRD4 (Cell Signaling Technology #13440), p-4EBP1 (Ser65, Cell Signaling Technology #9456) 4EBP1 (Cell Signaling Technology #9452), p-eIF4E (Ser209, Cell Signaling Technology #9741), eIF4E (Cell Signaling Technology #9742), p-S6K (Thr421/Ser424, Cell Signaling Technology #9204), S6K (Cell Signaling Technology #9202), Nestin (Santacruz Biotechnology #sc-23,927), SOX2 (Cell Signaling Technology #3579), GAPDH (Cell Signaling Technology #2118), Cyclophillin B (Cell Signaling Technology #43603), CD133 (BD Biosciences) and HRP-conjugated secondary antibodies (anti-Rabbit/Mouse, Jackson ImmunoResearch Laboratory).

### Statistical analysis

Each experiment was repeated at last an additional three times and the mean ± standard error values calculated. Statistical significance (*p*-value) was analyzed using two-tailed Student’s t-test or analysis of variance (ANOVA) and *p*-values > 0.05 considered significant. GraphPad Prism-V6 software was used to determine IC_50_ values and dose-response curves of inhibitors in NB cell lines. The Chou-Talalay combination index (CI) method was used to analyze synergy/interaction between inhibitors by using CalcuSyn software (Biosoft, UK). CI < 0.9 indicates synergism, 0.9–1.1 additivity and > 1.1 antagonism.

## Results

### Synergistic effects of JQ1 and temsirolimus on NB cell growth

We used small molecule inhibitors JQ1 and temsirolimus (TEM, hereafter) to target MYCN transcription (BET proteins) and mTOR signaling, respectively, [24, 25] in NB. Using the MTT assay, we first determined the IC_50_ of each inhibitor on cell viability of three non-MYCN- and three MYCN-amplified NB cell lines. Our results showed that as single agents, JQ1 and TEM inhibited NB cell growth with relatively lower IC_50_ values in MYCN-amplified NB cell lines (Table 1), suggesting superior efficacy of each inhibitor against MYCN-driven NB cell lines, compared to non-MYCN-amplified NB cell lines.

**Table 1:**
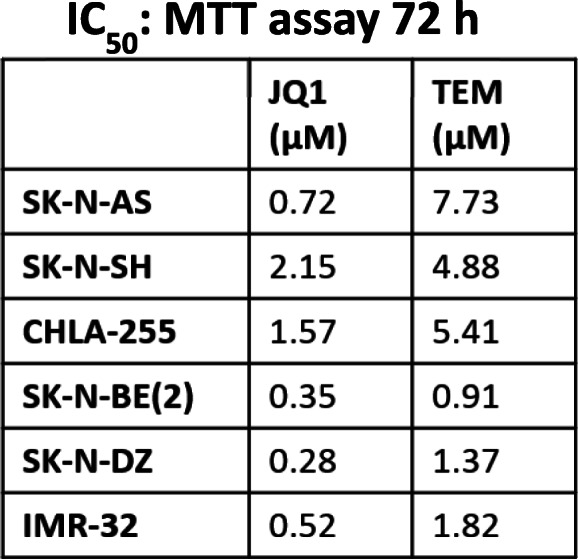
I3C50: MTT assay 72 h

**Fig. 1:**
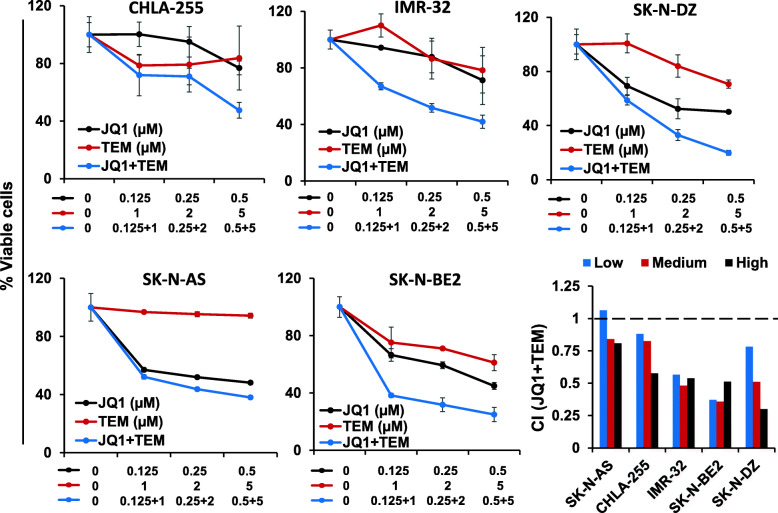
Synergistic effects of JQ1 and temsirolimus (TEM) on NB cell growth**.** Cell viability (MTT) assay showing dose-dependent growth effects of JQ1/TEM in NB cell lines at 72 h. Viable cells (%) is relative to DMSO-treated cells. Values represent mean ± SEM. Bar graphs show combination index (CI) analyses for the synergism of JQ1 and TEM in NB cell lines

We next tested the efficacy of combined JQ1/TEM to explore potential for synergistic growth inhibition on NB cells. NB cell lines, including three MYCN-amplified and two non-MYCN-amplified NB cell lines, were treated with increasing concentrations of JQ1 and TEM alone or in combination for 72 h. As shown in Fig. 1, co-treatment of JQ1/TEM significantly suppressed growth of all NB cell in a dose-dependent manner, compared with single agent treatment. Again, this co-treatment had greater on growth inhibition effects on MYCN-amplified cell lines, compared to non-MYCN-amplified cell line. The combination index (CI) analyses by Chou-Talalay method [26] confirmed that combination of JQ1/TEM had strong synergistic inhibitory effects on NB cell growth, with CI values ranging 0.3–0.8. These results suggested synergistic anti-NB potential of MYCN/mTOR inhibition.

### Co-treatment with JQ1 and TEM induces cell cycle arrest and apoptosis

**Fig. 2:**
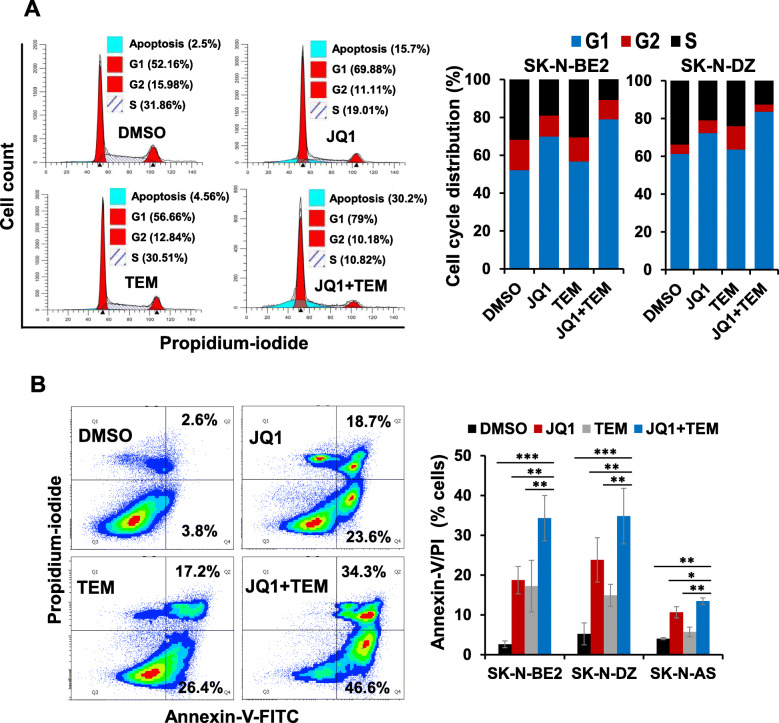
Combined effects of JQ1 and TEM on cell cycle arrest and apoptosis. **(a)** Representative flow cytometry plots show cell cycle distribution in SK-N-BE2 cells treated with JQ1 (0.5 μM) and TEM (2 μM) alone or combined for 24 h. On the right, graphs show the quantification of cell cycle distribution in two MYCN-amplified (SK-N-BE2, SK-N-DZ) NB cell lines treated with JQ1 (0.5 μM) and TEM (2 μM) alone or combined for 24 h. **(b)** Representative flow scatter diagrams show apoptosis induction in SK-N-BE2 cells treated with JQ1 (0.5 μM) and TEM (2 μM) alone or combined for 72 h. On the right, bar graphs show the quantification of Annexin-V/PI double positive apoptotic cells in two MYCN-amplified (SK-N-BE2, SK-N-DZ) and one non-MYCN-amplified (SK-N-AS) NB cell lines treated with JQ1 (0.5 μM) and TEM (2 μM) alone or combined for 72 h. Values, mean ± SEM. *p < 0.05; **p < 0.01; ***p < 0.001 (Student-*t*-test)

To determine combined effects of JQ1 and TEM on cell cycle and apoptosis, two highly MYCN-amplified NB (SK-N-BE2, SK-N-DZ) cell lines were treated with a suboptimal concentration of each inhibitor alone or in combination and subjected to cell cycle and apoptosis analyses using propidium-iodide and Annexin-V staining, respectively, followed by flow cytometry. The cell cycle analyses in both MYCN-amplified cell lines revealed that JQ1 and TEM alone slightly caused cell cycle arrest in G1 phase, while co-treatment with JQ1 and TEM drastically arrested the cells in G1 phase (Fig. 2a). The Annexin-V assay demonstrated that treatment with JQ1 or TEM alone increased the percentage of apoptotic cells. However, the co-treatment with JQ1 and TEM resulted in significant further induction of apoptosis in both NB lines (Fig. 2b) and showed consistency with the results of the MTT growth study. These results suggest that the combination of these two inhibitors suppresses growth and/or survival of MYCN-amplified NB cells in vitro.

### Co-treatment with JQ1 and TEM downregulates the expression of MYCN and mTOR signaling components

**Fig. 3:**
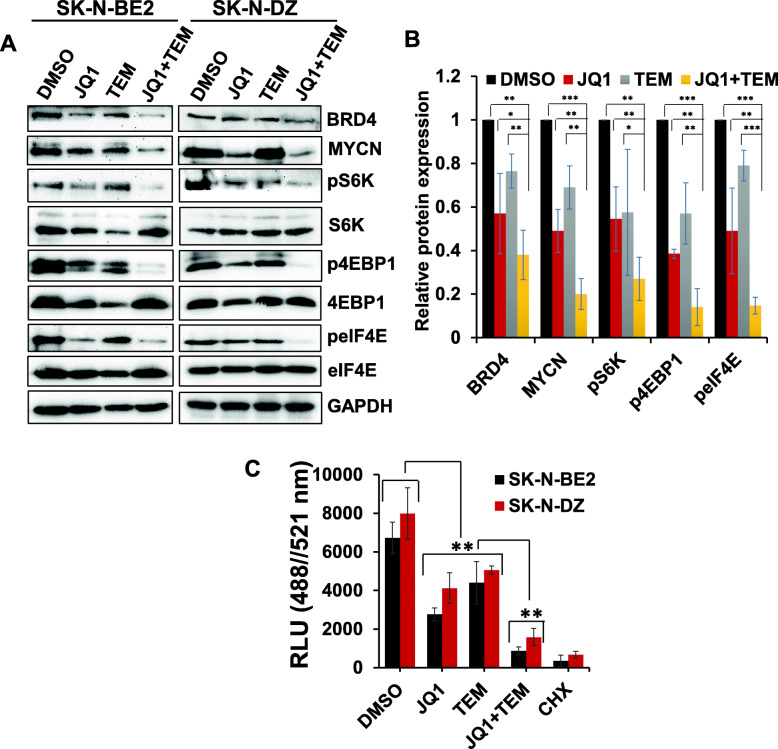
Combined effects of JQ1 and TEM on target pathways/molecules and global protein synthesis**. (a)** Western blot images for the expression of key components of MYCN/mTOR signaling in two MYCN-amplified NB cell lines following treatment with JQ1 (0.5 μM) and TEM (2 μM) alone and combined for 24 h. The original uncropped images of these blots are provided in the Additional File 1 (Fig. S1 and S2). **(b)** Bar graphs show the quantification of expression of key target proteins (shown in western blot images) relative to the control (DMSO) in the combined blots of SK-N-BE2 and SK-N-DZ cells after GAPDH (loading control) normalization using Image J software. The values represent the mean ± SEM of three replicates of blot. *p < 0.05; **p < 0.01; ***p < 0.001 (Student t test, vehicle/ or single agents vs. combination). **(c)** Overall protein synthesis measurement by OPP-incorporation following treatment with JQ1 (0.5 μM) and TEM (2 μM) alone or in combination for 24 h. CHX (50 μg/ml, 1 h) was used as a positive control for protein synthesis inhibition. Values represent mean ± SEM. **p < 0.01 (Student-*t*-test)

To establish the molecular mechanism(s) associated with JQ1/TEM anti-NB activity, we examined the expression/activation of key components of MYCN and mTOR signaling pathways by western blotting in SK-N-BE2 and SK-N-DZ cell lines. Single agent treatments of MYCN-amplified cells with JQ1 or TEM significantly suppressed expression of MYCN and BET protein BRD4 and downregulated the levels of phosphorylated/activated signaling proteins (p-S6K, p-4EBP1, and p-eIF4E) of the mTOR (translation) pathway (Fig. 3a and b**)**. Co-treatment with JQ1 and TEM further downregulated the expression of the above mentioned mTOR signaling components, as well as MYCN expression, compared with single agent treatments. These data suggest that concurrent inhibition of MYCN transcription and mTOR signaling cooperatively suppresses the protein synthesis pathway, justifying why this combined inhibition exerts the greatest antitumor effects in MYCN-amplified NB.

### Co-treatment with JQ1 and TEM inhibits global protein synthesis

Because of the key role of MYCN/mTOR signaling in controlling global protein synthesis, we further tested whether the inhibition of MYCN/mTOR represented a global blockage of protein synthesis. To this end, we performed protein synthesis assay using a robust chemical method based on a cell permeable analogue of puromycin, O-Propargyl-puromycin (OPP), in SK-N-BE2 and SK-N-DZ cell lines treated with JQ1 and TEM alone or in combination. Incorporation of OPP to nascent polypeptide chains can be labeled via copper catalyzed click-chemistry using 5-FAMAzide in order to quantify total protein synthesis. In this assay, we used cycloheximide as the positive control for protein synthesis inhibition. This fluorescence-based assay displayed high protein synthesis in control solvent (DMSO)-treated cells and strong inhibition of protein synthesis when blocked with cycloheximide (Fig. 3c). Although treatments of JQ1 and TEM alone showed strong inhibitory effects on protein synthesis, the combination of these two caused significant further reduction in total protein synthesis, resulting in the lowest fluorescent signal in both NB cell lines and suggesting a synergistic effect of the MYCN and mTOR targeting on global protein synthesis.

### Combined effect of JQ1 and TEM on neurosphere formation

**Fig. 4:**
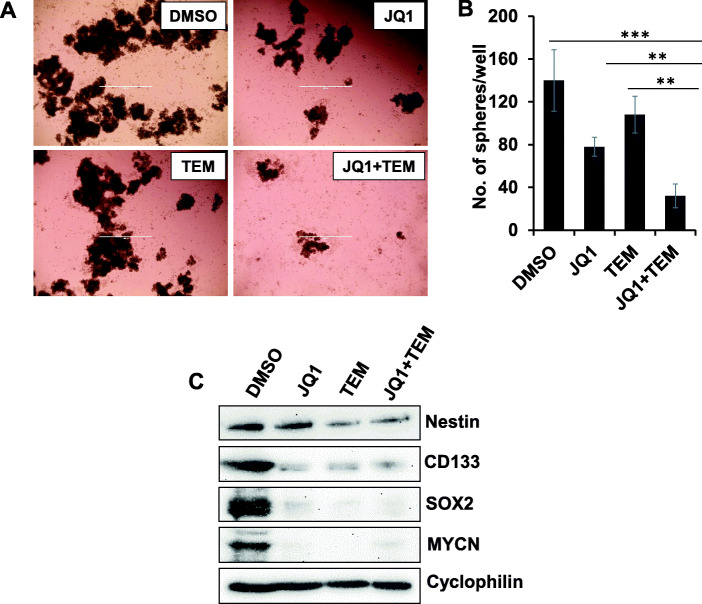
Combined effects of JQ1 and TEM on neurosphere formation. **(a)** Representative micrograph of spheres of SK-N-BE2 cells with the treatment of control-(DMSO) or JQ1 (0.5 μM) and TEM (2 μM) alone or combined for 7 days. Scale bar, 1000 μm. **(b)** Quantification of the number of neurospheres following treatments. The values represent mean ± SEM. **p < 0.01; ***p < 0.001 (Student-*t*-test). **(c)** Western blot analysis for the expression of neural stem cell markers following treatment of neurospheres with JQ1 (0.5 μM) and TEM (2 μM) alone or combined for three days. Cyclophilin was used as the loading control in this analysis. The original uncropped images of these blots are provided in the Additional File 1 (Fig. S3)

We next investigated the effect of JQ1 and TEM, alone or combined, in a neurosphere model of MYCN-amplified NB cells. Figure 4a shows micrographs of sphere formation in SK-N-BE2 cells. Treatment with JQ1 or TEM alone significantly inhibited sphere formation, with further reduction of sphere formation when both were combined (Fig. 4b). We further tested the effects of JQ1 and TEM on the expression of neural stem cell markers (Nestin, CD133, SOX2) in SK-N-BE2 spheres by western blot analysis. We observed that JQ1 and TEM, either alone or combined, strongly inhibited the expression of these stem cell markers and inhibited MYCN expression (Fig. 4c). These data suggest that combined inhibition of MYC transcription and mTOR signaling has anti-tumor effects on neurospheres and associated stem cell markers.

### JQ1 and TEM chemosensitizes NB cells

**Fig. 5:**
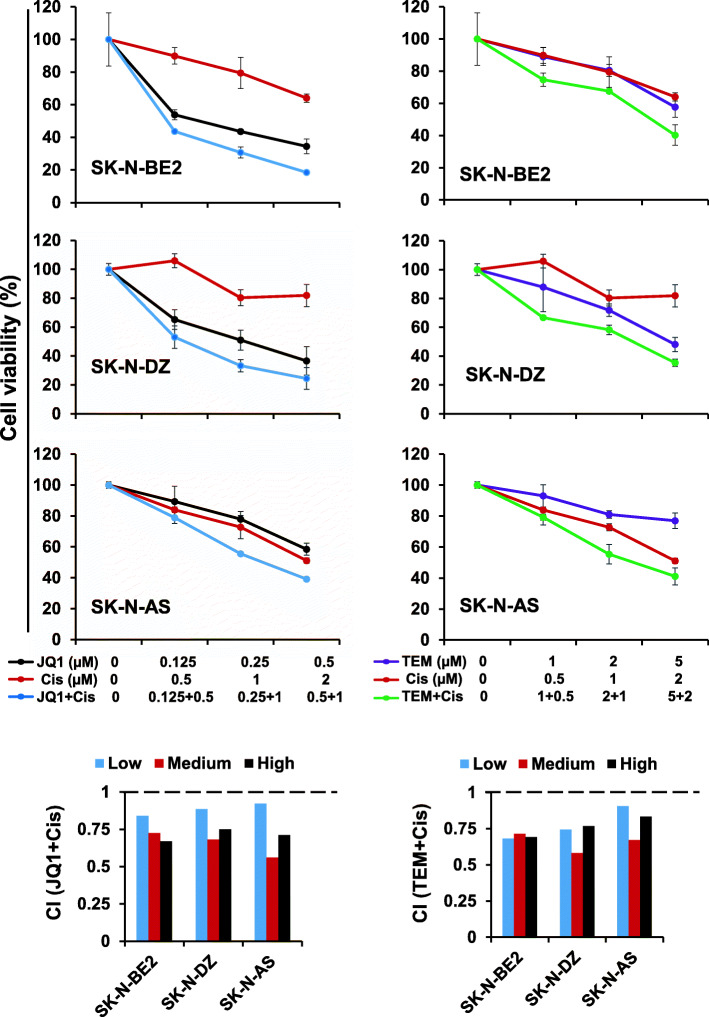
JQ1 and TEM chemosensitize NB cells. MTT results showing the dose-dependent effects of JQ1 and TEM alone or combined with cisplatin (Cis) chemotherapy in NB cell lines at 72 h, as indicated. The values represent the means ± SEM. Bar graphs show combination index (CI) analyses for the synergism between JQ1/Cis or TEM/Cis in NB cell lines

Given the limited success of current therapies, we next sought to determine whether JQ1 or TEM could enhance the anti-NB efficacy of chemotherapy by sensitizing NB cells. Cisplatin is one of the most common chemotherapeutic drugs in the treatment of NB patients. To evaluate the enhanced efficacy of inhibitors on cisplatin-mediated NB cytotoxicity, we treated NB (SK-N-AS, SK-N-BE2, SK-N-DZ) cell lines with cisplatin and either JQ1, TEM, or the combination cisplatin alone or in combination, in stepwise doses. At 72 h we determined cell growth using the MTT assay. Results shown in Fig. 5 clearly show that co-treatment of NB cell lines with inhibitors (JQ1 or TEM) and cisplatin significantly inhibited cell growth in a dose-dependent manner, compared to cisplatin and inhibitors alone. Combination index analyses further show that JQ1 or TEM synergistically increased the cytotoxicity of cisplatin in all NB cell lines tested. Of these combinations, JQ1 demonstrated a significantly greater efficacy in enhancing cisplatin-mediated NB cytotoxicity. Results also indicated a higher sensitivity of MYCN-amplified NB cells to these combined treatments, compared to non-MYCN-amplified SK-N-AS cells. In summary, these data show that JQ1 or TEM either combined together or individually combined with cisplatin chemotherapy, synergistically inhibits NB cell growth.

### Synergistic anti-cancer efficacies of OTX-015 and TEM in NB cells

**Fig. 6:**
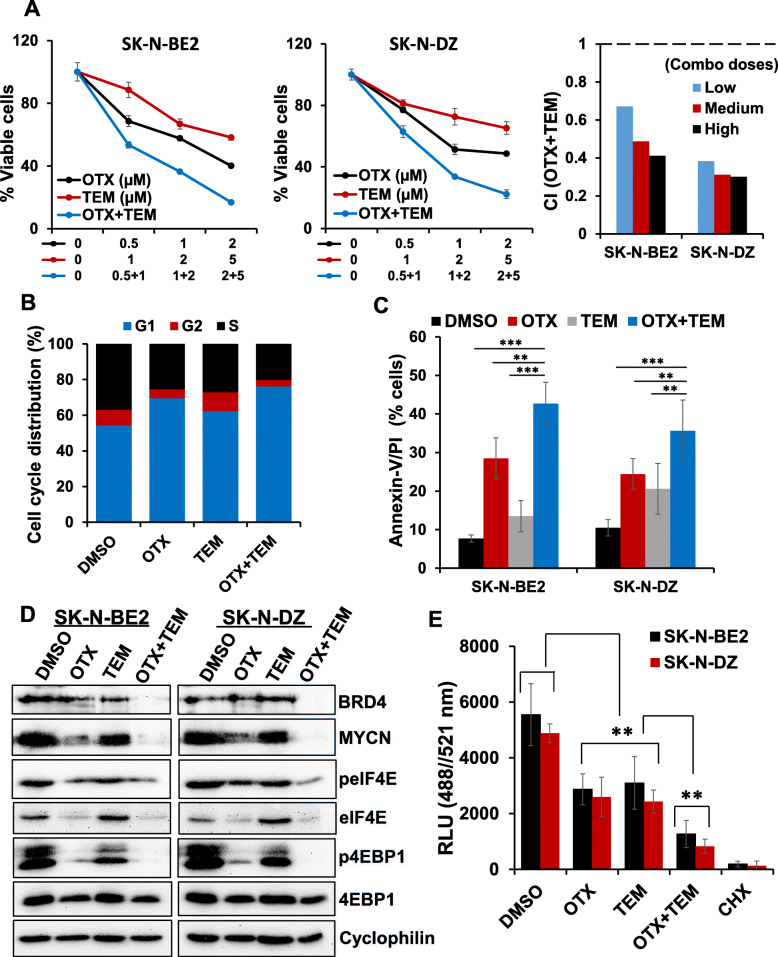
Synergistic effects of OTX-015 (OTX) and TEM on NB cell growth. **(a)** Cell viability (MTT) assay showing dose-dependent growth effects of OTX/TEM in two MYCN-amplified NB cell lines at 72 h. Viable cells (%) is relative to DMSO-treated cells. Values represent mean ± SEM. Bar graphs show combination index (CI) analyses for the synergism of OTX and TEM in NB cell lines. **(b)** Analysis of cell cycle distribution in SK-N-BE2 cells treated with OTX (1 μM) and TEM (2 μM) alone or combined for 24 h. **(c)** Quantification of Annexin-V/PI positive apoptotic cells in two MYCN-amplified (SK-N-BE2, SK-N-DZ) NB cell lines treated with OTX (1 μM) and TEM (2 μM) alone or combined for 72 h. Values, mean ± SEM. **p < 0.01; ***p < 0.001 (Student-*t*-test). **(d)** Western blot images for the expression of key components of MYCN/mTOR signaling in two MYCN-amplified NB cell lines following treatment with OTX (1 μM) and TEM (2 μM) alone and combined for 24 h. Cyclophilin was used as the loading control in these analyses. The original uncropped images of these blots are provided in the Additional File 1 (Fig. S4). **(e)** Global protein synthesis measurement by OPP-incorporation following treatment with OTX (1 μM) and TEM (2 μM) alone or in combination for 24 h. CHX (50 μg/ml, 1 h) was used as a positive control for protein synthesis inhibition. Values represent mean ± SEM. **p < 0.01

In previous experiments, the rationale for using JQ1 was its advantages over other BET inhibitors in preclinical cancer studies. Preclinical studies with JQ1 offered a great opportunity to better understand the biology of BET proteins and validate these proteins as the anti-cancer targets [14, 24]. In addition, JQ1 has been shown to efficiently target MYC/MYCN transcription in pediatric NB and medulloblastoma [15–17]. However, JQ1 is not being considered in clinical trials because of its short half-life [24]. As the proof of concept that if the combined inhibition of BET protein and mTOR signaling can be translated into the clinic, we also utilized a clinically relevant BET protein inhibitor OTX-015 (OTX, hereafter) which is currently in clinical trials for several advanced cancers [14]. Using previously reported doses of OTX in NB cell lines [16], we tested its combination efficacy with TEM in two MYCN-amplified NB cell lines. As shown in Fig. 6a, co-treatment of OTX and TEM significantly suppressed growth of NB cell lines in a dose-dependent manner, compared to single agents alone. CI analyses further confirmed that combination of OTX and TEM had a highly synergistic inhibitory effect on NB cell growth with a CI value below 0.7. The results from cell cycle (PI staining) and apoptosis (Annexin-V/PI staining) analyses in NB cells showed that the combination significantly induced G1 cell cycle arrest and apoptosis (Fig. 6b and c), compared to single agents. Our western blot results showed that compared to single agent treatments, combination of OTX and TEM significantly suppressed expression of MYCN and downregulated the levels of key downstream targets (phosphorylated-4EBP1/eIF4E) of the mTOR pathway (Fig. 6d). The results from global protein synthesis investigation using OPP-based assay, further demonstrated synergistic inhibition of general protein synthesis rate by OTX and TEM, compared to single agents (Fig. 6e). These results consistently suggested the importance of co-targeting BET protein and mTOR signaling in NB.

## Discussion

Despite the availability of intensive multimodal therapy, the prognosis for patients with MYCN-amplified NB remains extremely poor [4]. Since MYCN is not directly druggable, indirectly inhibiting it by targeting upstream or downstream components of the MYCN pathway that can regulate its oncogenic activities might be the ideal option [10, 12]. As a transcription factor, MYCN has tight control over global transcription and translation [9]. Aberrant activation of several oncogenic pathways, including MYC pathway, is a hallmark of many childhood cancers, including NB [10]. MTOR signaling is one of the key oncogenic pathways which is often deregulated in MYC-driven cancers, including NB [20]. Moreover, it has been shown that MYC proteins and the mTOR pathway cooperate with each other at the transcription and translation levels, respectively, to elevate overall protein synthesis rate, leading to increased cell proliferation and cancer progression [21, 22, 27]. These pathways, either working individually or together, can cause cancer progression. However, inhibition of one pathway alone will often result in minor to moderate anti-tumor activity, suggesting concurrent targeting of MYCN transcription and mTOR signaling is important strategy to enhance efficacy. Here, using small molecule therapeutics, we show that combined inhibition of MYCN transcription by BET inhibitors (JQ1/OTX) and mTOR signaling by TEM, synergistically inhibited NB cell growth and survival. The combined activity was more potent than inhibition of either single pathway.

BET protein inhibitors exert biological effects by competitively binding to the acetyl-lysine pockets and displacing BET proteins from chromatin, leading to the repression of oncogene transcription. BET protein inhibitors have been shown to target MYC/MYCN transcription in several cancers, including NB, demonstrating their potential as preclinical anticancer agents [13–17]. In comparison, mTOR inhibitors, another class of anticancer agents, exert their inhibitory effects by blocking the activation of mTOR signaling, leading to inhibited translation of proteins, including MYCN protein [21, 25]. Several inhibitors of BET protein and mTOR signaling either alone or in combination with other target agents, are in multiple clinical trials in patients with advanced cancers [14, 25]. In this study, we used JQ1, a potent preclinical BET protein inhibitor, as a proof of concept for targeting MYCN transcription [16]. Since JQ1 is not being tested in clinical trials due to its short half-life [28], in parallel, we used the clinically relevant BET protein inhibitor OTX-015 as an alternative agent to target MYCN. We confirmed that JQ1 and OTX-015 have similar anti-NB efficacy in our proof-of-concept study. To target mTOR signaling we used the clinically relevant mTOR inhibitor TEM [25], an FDA approved drug for the treatment of renal cell carcinoma. These inhibitors have demonstrated promising antitumor activities against multiple solid and hematological cancers [14, 25]. Here, we identify a synergistic interaction between BET and mTOR inhibitors to induce antitumor effects in NB cells. In addition, our evidence of cell cycle arrest, induction of apoptosis and inhibition of NB neurospheres further reinforce the clinical relevance of our combination approach.

In addition, our data provide insights into the molecular mechanism(s) underlying the antitumor efficacy of JQ1/TEM we observed in NB. We show that co-treatment with JQ1 and TEM synergistically reduce the expression of key component proteins of the activated mTOR translation pathway, along with suppression of MYCN and BRD4 (a key member of BET family proteins) protein expression in NB cells. We further show that this co-inhibition of MYCN/mTOR by JQ1/TEM results in significant blockade of global protein synthesis in NB cells. These observations in NB are consistent with our previous study in medulloblastoma where we have shown similar combined activity of JQ1/TEM on MYC/mTOR targets, leading to inhibition of global protein synthesis. Overall, these findings suggest a synergistic and cooperative activity of JQ1/TEM on MYCN and mTOR pathways in aggressive NB.

While our data show that although individual or concomitant inhibition of BET/mTOR has greater antitumor efficacies against MYCN-amplified NB cells, the combination also efficiently inhibits cell growth and induces apoptosis in non-MYCN-amplified NB cells, pointing to a broader relevance of our combined approach in NB. Because non-MYCN-amplified NB cell lines, including SK-N-AS, express MYC [29] which is a target for combined BET/mTOR in medulloblastoma cells [22]. MYC may be a secondary target for these inhibitors in the context of NB.

In NB and other cancers, MYCN and mTOR signaling have been shown to play key roles in cancer stem cells and contribute to relapse and drug-resistance. NB cells express neural stem cell markers such as CD133 and Nestin and have the ability to form neurospheres [30–33]. Our initial findings with the anti-NB efficacy of MYCN/mTOR inhibition on neurospheres and stem cell markers indicate that individual or combined inhibition of MYCN/mTOR might target cancer stem cells - potentially minimizing recurrence of NB. Mounting evidence suggest that increased protein synthesis in cells not only correlates with increased cell proliferation/survival, but also often involves in stem cell fate, including neural stem cell markers [34, 35]. In line with these, our data is consistent that cooperative inhibition of MYCN/mTOR by proposed drugs downregulates the expression/activation of MYCN/mTOR key targets and inhibits global protein synthesis, thereby enables cell growth/cell cycle arrest, apoptosis and inhibits neurosphere formation/stem cell markers.

Since overexpression or amplification of MYC/MYCN and activation of mTOR signaling are often associated with chemoresistance in many cancers including NB [36–38], we also wanted to see whether inhibition of these signaling pathways helps to chemosensitize NB cells. Our results revealed that both JQ1 and TEM significantly enhanced cisplatin-induced growth inhibition in MB cells, suggesting that inhibition of MYCN/mTOR signaling not only inhibits cell proliferation and survival of NB cells, but also sensitizes NB cells to chemotherapy. The exact mechanism of synergy between these inhibitors and cisplatin requires further investigation. However, activation of both MYC and PI3K-mTOR signaling pathways have been shown to be associated with platinum-based therapy resistance [39–41]. The cytotoxicity of cisplatin involves the damaging of DNA replication/repair mechanisms. Similarly, targeting MYC and mTOR signaling have been shown to induce DNA damage response and contribute susceptibility to cisplatin-induced cell death. Therefore, co-induction of DNA damage by cisplatin and JQ1 or TEM could be the potential mechanism of observed synergy.

In clinical trials studies, TEM alone or in combination with other clinical agents, such as chemotherapeutic drugs temzilomide and irrinotican, has shown no significant benefit for relapsed NB patients [42, 43]. This suggests further exploration of combining TEM with other targeted therapies. Our data indicate that one of potential combinatorial target could be the inhibition of BET proteins. Since inhibitors of BET proteins, including OTX and structurally similar inhibitors to JQ1, are currently in clinical trials for several cancers, it is more likely that combination of TEM with BET inhibitors can be translated in clinic for NB and other MYC/MYCN-driven cancers therapies.

## Conclusions

Our study demonstrates that targeting dysregulated protein synthesis pathway by pharmacologic dual-inhibition of MYCN transcription (by BET protein inhibition) and mTOR signaling has significant preclinical anti-NB efficacies in inducing cell growth inhibition, cell cycle arrest and apoptosis in vitro. Combination of JQ1 or OTX with TEM synergistically inhibited global protein synthesis by downregulating the key components and downstream targets of MYCN/mTOR signaling. Thus, this study is the first to demonstrate synergy in the combined inhibition of BET protein and mTOR signaling in NB. Our study also revealed that inhibition of MYCN/mTOR not only inhibits cell growth and survival in NB cells, but also chemosensitizes NB cells. Beyond NB, this therapeutic approach can be of broader relevance for therapy of MYC/MYCN-addicted cancers, as we have previously shown a synergistic antitumor efficacy of BET/mTOR inhibitors in MYC-driven medulloblastoma [22]. While further studies using appropriate in vivo models are needed to evaluate this combination, our study highlights a basis for considering this combination approach as a new therapy for NB.

## Supplementary Information

**Additional file 1.**

## Data Availability

All data generated or analyzed during this study are included in this article.

## Abbreviations

BET: Bromodomain extra-terminal
CI: Combination index
FBS: Fetal bovine serum
h: Hour
IC_50_: Inhibitory concentration of inhibitor with 50% inhibition
NB: Neuroblastoma
mTOR: Mammalian Target of Rapamycin
MTT: 3-(4,5-dimethylthiazol-2-yl)-2,5-diphenyltetrazolium bromide;
MYC: v-myc avian mylocytomatosis viral oncogene homolog
MYCN: v-myc avian mylocytomatosis viral oncogene neuroblastoma homolog
OPP: O-Propargyl-puromycin
OTX: OTX-015
TEM: Temsirolimus
μM: Micromolar
