# Supplementary material for: Synergistic efficacy of inhibiting MYCN and mTOR signaling against neuroblastoma
Source: BMC Cancer. 2021 Sep 26;21:1061. doi: 10.1186/s12885-021-08782-9 (PMC8474810; doi:10.1186/s12885-021-08782-9)
Supplement: Supplementary file 1 — Additional file 1. [file 12885_2021_8782_MOESM1_ESM.docx]

**Fig. S1.** Below are the original uncropped blot images in SK-N-BE2 cells shown in Fig. 4a of the main manuscript. The membranes were cut (based on molecular weight of indicated proteins), probed with specific antibodies, and imaged using an ECL imager. The cropped images are highlighted with rectangle black lines in each blot.

*Fig.S1 continued to the next page*

**Fig. S2.** Below are the original uncropped blot images in SK-N-DZ cells shown in Fig. 4a of the main manuscript. The membranes were cut (based on molecular weight of indicated proteins), probed with specific antibodies, and imaged using an ECL imager. The cropped images are highlighted with rectangle black lines in each blot.

*Fig.S2 continued to the next page*

**Fig. S3.** Below are the original uncropped blot images in SK-N-BE2 spheres shown in Fig. 5C of the main manuscript. The membranes were cut (based on molecular weight of indicated proteins), probed with specific antibodies, and imaged using an ECL imager. The cropped images are highlighted with rectangle black lines in each blot.

**Fig. S4.** Below are the original uncropped blot images in SK-N-BE2 and SK-N-DZ cells shown in Fig. 6D of the main manuscript. The membranes were cut (based on molecular weight of indicated proteins), probed with specific antibodies, and imaged using an ECL imager. The cropped images are highlighted with rectangle black lines in each blot.

*Fig.S4 continued to the next page*
